# BIM Based Virtual Environment for Fire Emergency Evacuation

**DOI:** 10.1155/2014/589016

**Published:** 2014-08-13

**Authors:** Bin Wang, Haijiang Li, Yacine Rezgui, Alex Bradley, Hoang N. Ong

**Affiliations:** School of Engineering, Cardiff University, Queens Building, The Parade, Cardiff CF24 3AA, UK

## Abstract

Recent building emergency management research has highlighted the need for the effective utilization of dynamically changing building information. BIM (building information modelling) can play a significant role in this process due to its comprehensive and standardized data format and integrated process. This paper introduces a BIM based virtual environment supported by virtual reality (VR) and a serious game engine to address several key issues for building emergency management, for example, timely two-way information updating and better emergency awareness training. The focus of this paper lies on how to utilize BIM as a comprehensive building information provider to work with virtual reality technologies to build an adaptable immersive serious game environment to provide real-time fire evacuation guidance. The innovation lies on the seamless integration between BIM and a serious game based virtual reality (VR) environment aiming at practical problem solving by leveraging state-of-the-art computing technologies. The system has been tested for its robustness and functionality against the development requirements, and the results showed promising potential to support more effective emergency management.

## 1. Introduction

Building emergency management can be generally illustrated as an integrated scientific methodology to provide reasonable solutions for human safety in extreme environments [1] and this is particularly significant in addressing the regrettably common occurrence of fire disaster, which directly relates to the lives and property safety of all occupants in the building [1, 2]. Recent relevant research has identified that the cause of a high proportion of emergency casualties has a direct link with the delayed evacuation service of the facility [3, 4], which can be caused by the lack of real-time two-way information updates; building users (and visitors) in an emergency situation cannot get the real-time evacuation route, while the external control centres lack real-time situation updates, for example, the real-time location of building users.

Traditionally, fire emergency management utilizes fire drills or experiments to enhance fire emergency planning. However, there has been little discussion about awareness of the safety situation in a fire drill and the resource costs for emergency experiments, which influence the validity and generalizability of their results [5]. Moreover, the abovementioned traditional measures can only provide solutions after the completion of building design; hence, it is difficult to detect the conflicts (to fire emergency evacuation plan in practice) early during the design stage, and the follow-on modification process (to address fire emergency issues) would be very time consuming and costing.

BIM promotes integration and collaboration which allows applications at different stages of the building life cycle to be effectively “linked” through the shared information, but a significant number of current BIM developments have been directed to design collaboration and the subsequent economic benefits for design professionals; dedicated BIM based solutions for building emergency management are relatively less focused [6]. BIM can play a significant role in providing real-time and accurate building information under an emergent situation due to its comprehensive and standardized data format and integrated process.

Although several recent research studies on fire emergency management have been trying to embed human behaviour modelling into building emergency management, there is still a lack of studies that can adequately and precisely represent human behaviour in emergent situation [7] and conduct effective information interaction between the building and the building user [8]. Kobes et al. proposed to use virtual games to get a real-time observation of human behaviour in a fire as video recording of real fire evacuations are rare [9].

This paper introduces a framework that utilizes BIM as a building information provider to work with serious game technologies to build an adaptable virtual reality environment with the purpose of enhancing fire evacuation plans throughout the building life cycle. The focus lies on how to utilize updated building information with virtual reality technology to provide real-time fire evacuation guidance. The developed virtual reality environment can also be utilized during the design stage by leveraging the engagement of a wider audience, such as general end-users, who are the genuine users of a building and play a vital role in assisting professionals to achieve satisfactory building emergency designs and follow-on services.

The following contents are organized as follows.  Section 2 explains the related work; Section 3 shows the design and implementation for a BIM-VE (virtual environment), including a two-way information channel between the building information model and the serious game environments, supporting computing hardware, and the overall server/client based infrastructure and information flow.  Section 4 explains the case application for fire evacuation, including algorithm development and implementation for BIM based dynamic emergency route planning on multiple platforms during different stages of building life cycle, and dynamic scenarios generation with building semantic information for fire emergency training. System testing and evaluation are detailed in Section 5. The discussion follows and the conclusion and future work are given at the end.

## 2. Related Work

Building emergency management concerns several main aspects, such as emergency preplanning, emergency psychological human behaviour, and timely information communication [1, 10]. Emergency preplanning is an action plan devised as a precautionary measure before any disaster and is activated in response to major incidents only. The normal approach to address emergency preplanning includes preplanning drills and digital preplanning [1, 5]. The preplanning drills are to record the behaviour of participants (as evacuees), which usually includes the completion of a postevacuation questionnaire to supplement and supply the results that are hard to be observed, such as perception of emergency cues during the drills. Several research projects utilized this approach to study the behaviour of store shoppers [11, 12], with some interesting findings, such as the observation that some exits were not used since there was no staff to direct there and some evacuees showed a reluctance to pass disabled evacuees, which was one of the reasons for an increase in evacuation time. Although taking recordings of drill participants, followed by a questionnaire analysis, is the most common method to support the emergency preplanning, it often covers only singular aspects of human behaviour. Also, the contents of a questionnaire are sometimes not necessarily useful because participants know they are not in a dangerous situation and therefore suffer no cognitive emergency stress. In addition, real world emergency drills cannot be conducted regularly and the participants are also limited to the people who are in the building during the emergency drill [5].

In terms of digital preplanning, an emergency preplanning semantic retrieval system for facility managers was implemented to retrieve related knowledge from relevant management documents [13]. Yan built a core library which can be used to modify, query and match, judge and evaluate, and classify and analyse the preplans. Through this digitalized emergency preplanning, the end-users can create an evacuation scheme and monitor the evacuation process in real time [10]. Nonetheless, the abovementioned digitalized preplans heavily rely on the complicated database which sometimes can be difficult to be maintained and updated to a satisfactory level to retrieve meaningful results for the end-users. Rüppel and Stuebbe combined building information and indoor navigation systems on mobile devices to improve fire emergency plans and route finding for complex buildings [14]. However, the building information for such a system is static and limited, which means it cannot dynamically change the shortest path displayed according to the constantly changing situation during a fire emergency. Lastly, this system was limited to specific mobile devices used by fire fighters rather than common mobile devices such as smart mobile phones utilized by general end-users of the building.

Incident analysis has revealed that there is a link between a delayed evacuation and a high number of fire deaths and injuries, especially in residential and high-rising buildings [15]. One of the most widely known examples illustrating this link is the 2001 terrorist attack on the World Trade Centre (WTC). Several methods were employed for information gathering such as first-person accounts taken from newspapers, radio and television programmes, e-mail exchanges, and a variety of websites, questionnaires, telephone interviews, and face-to-face interviews [16]. The comprehensive process of data collection mentioned above consumed a huge amount of time and money and is restricted to this specific scenario. According to Kobes et al., employing a serious game to collect information on human behaviour in a simulated fire disaster could be an efficient method to save time and money [9].

Recent computing developments have enabled computer gaming systems to be utilized for building emergency management [6]. A BIM based serious game system was designed to explore human behaviour during a fire emergency [8], but the file system they used to transfer building information to the game environment is semiautomatic. If the participants in the virtual experiment adapt and get familiar with the scenarios, they have to manually stop the serious game and change the scenario, which makes the participants lose focus. Other researchers are working on the combination of human emergency behaviour with simulation technology [7, 17]. Li et al. proposed a prototype of a behaviour based human motion simulation for fire evacuation procedures [17]. Ren et al. developed a virtual system with spreading fire and smoke to simulate a fire evacuation based on the interaction between AI and the virtual fire environment [7]. However, questions have been raised about how to precisely represent emergency behaviour to simulate a fire evacuation [5]. According to Sime [18], some human behaviours during an evacuation have not been sufficiently understood and require further study to build a connection between the fire evacuation and fire safety engineering. Lin et al. enhanced emergency path planning by utilising the semantic information from IFC and the Fast Marching Method [19]. But the geometric and semantic information defined in the IFC have to be imported into the specific virtual environment manually, which is time consuming and ineffective.

## 3. BIM Based Virtual Environment (BIM-VE)

### 3.1. Virtual Reality Equipment in Cardiff University

Virtual environments (VEs) are three-dimensional, computer generated environments that occur in the real world but can be manipulated by the end-users [20]. The virtual reality technology that can generate high quality VEs has been introduced to many fields to assist military training, industry product design, and serious academic investigation. Its ability to allow end-users to experience a range of threatening or dangerous situations without physical harm provides many potential benefits. Guo et al. used game and virtual reality technologies to develop a safety training platform to improve the safety of construction plant operations. They utilised Wii controllers to provide trainees with the hands-on practical tools that allowed them to conduct some construction plant tasks without physical danger in a virtual environment. Through this safety training, the trainees can understand operating processes, improve collaboration among operators, and identify safety problems on the construction site [21]. Similarly, Harris and Morgenthaler developed virtual prototyping simulation tools to demonstrate ground processing, depict real-time visualization of design, and plan aerospace missions in a 3D immersive visualization environment (IVE) [22]. Rüppel and Schatz have begun to design a BIM based serious game for fire safety evacuation simulations in the Darmstadt CES-lab [8]. Darmstadt CES-lab is a virtual reality lab that provides hardware support to generate virtual environments in the sense of an immersive system. As for the software component, a BIM-game engine is being built that utilizes a file based information interchange mechanism to transfer building information and simplify physical interactions between objects. However, the building information transferred via the BIM-game engine is semi-automatic and cannot be updated in real-time. With augmented reality, Koch et al. presented a conceptual framework that uses the camera of a mobile device to recognize natural markers (e.g., exit signs), which can mix the virtual and real environment to provide facility maintenance support [23].

**Figure 1:**
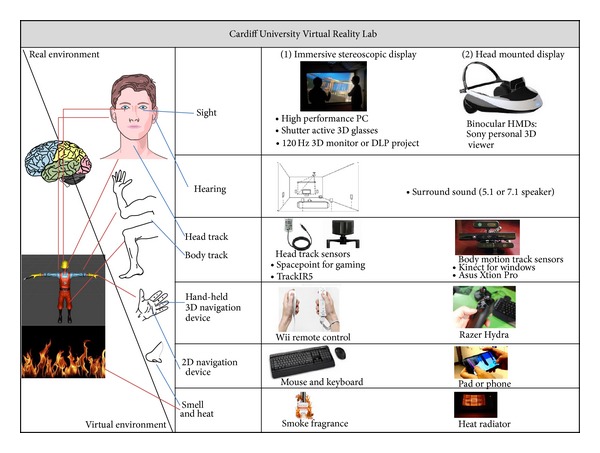
Existing and planned virtual reality equipment in Cardiff VR Lab.

The quality of a VE's representation varies greatly, but it is agreed that the more accurate details a VE can map with the real, the more immersive effects the end-users can feel. Suitable hardware for the expression of a VE has been implemented in Cardiff Virtual Reality Lab, which has been established at the Engineering School of Cardiff University. Cardiff VR Lab comes with an efficient VE in the case of an immersive system. It provides a natural interface between humans and computers by artificially imitating the way humans interact with their physical environment. The virtual reality equipment in the lab intends to enhance the main senses of the end-user which include sight, hearing, feeling, touching, and smell, utilising both the output of sensory information and input commands from the user. The output equipment (Figure 1) includes an immersive stereoscopic display, head mounted display (HMD), and a surround sound system. Input facilities include motion tracking sensors such as head tracking sensors and body tracking sensors, hand-held 3D navigation devices such as a Wii remote control or Razer Hydra joystick, a 2D navigation device such as a mouse and keyboard or a touch pad/phone, and devices that mimic the physical environment such as smoke fragrance and heat radiator.

In order for the VE to mimic the real environment, it must be able to integrate the sensory output of the environment to the real actions (navigation) of the end-users. It should couple the visual output (for perception of fire, tracking of moving characters, distance judging, space searching, and building environment estimation) and auditory output (for emergency recognition and localization) with the end-users' navigation (first person view, third person view, fly-through view, and manipulation of building objects). Ideally, the end-users in the VE are fully immersed and feel they are actually “present” in fire, to prevent them from making unrealistic decisions due to a lack of physical feelings in the VE [20, 24]. To achieve the above, the following capabilities are needed to immerse the end-users into the VEs:

- high-resolution, 24-bit colour, flicker- and ghosting-free, 3D stereoscopic display to maximize the visual stimulus of fire conditions (i.e., the 3D projector for group views and the head mounted display (HMD) for better personal view),
- sound effects which should surround end-users to allow them to recognize and locate the emergency by appreciable fire factors such as shouting or crying people, fire alarm, and noise of the roaring fire,
- motion tracking to use multiple parts of his body (e.g., hands, head, and legs) to interact with the VE to enhance the end-user's physical feeling; it also allows the display of a realistic avatar based on the profile of each end-user, which can closely imitate their actual movements and physical dimensions,
- navigation devices that allow accurate direction pointing, fly-through, or walk-through in the environment and manipulation with building object like extinguisher,
- light source based rendering and photorealistic textures,
- visual consistency (the building elements' position and appearance are predictable like in a real environment),
- decreased disturbance from virtual and real world.

### 3.2. Overall System Architecture

**Figure 2:**
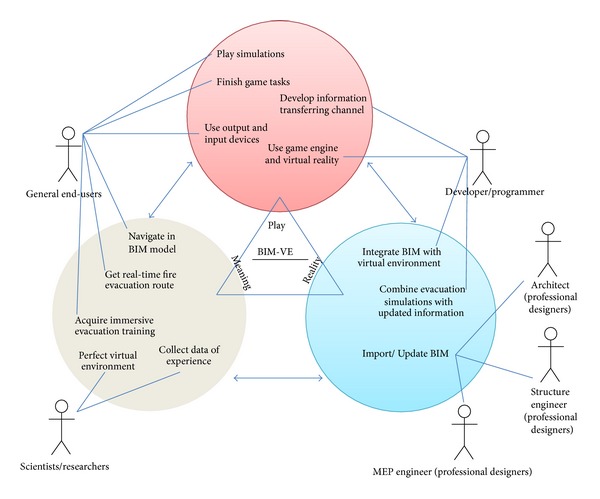
TGD based framework to develop BIM based VE.

The approach of triadic game design (TGD) proposed by Harteveld [25] indicates that a virtual environment designer has to balance three independent worlds during the design process, that is, the world of reality, meaning, and play. The world of “reality” deals with how the virtual environment is connected to the physical world; the world of “meaning” focuses on the type of value that needs to be achieved; the world of “play” concerns the methods used to reach the objectives in the world of “meaning.” The BIM based VE is shown in Figure 2, which illustrates what use-cases and which actors are involved and how they can be put together to achieve a balanced virtual environment.

Although video games have been available for almost thirty years, nonprofessional programmers have only recently been able to modify the virtual environments within games, because the editors used to modify them (game engine) have become very sophisticated but conversely easy to use [26]. As one of the most famous game engines, Unity3D is characterized by its multiple-platform system support and is available in free and commercial versions. With the Unity3D game engine, games can be exported as standalone applications for OSX and MS Windows, for consoles such as XBox and Wii, and for smartphones running iOS, Android, Blackberry, and Windows. More importantly, it supports web applets for online use, which can decrease the size of a game and promote its spread. Therefore, the Unity3D game engine can develop serious games for a wide range of end-users to gather measurable and quantifiable information for research (i.e., enhance quantitative research results). The Unity3D game engine provides very high quality shader, rendered textures, and interface to work with other platforms (including BIM modelling tools and virtual reality equipment) to make the virtual environment in the serious game comparable to the real and can be associated with an interpretative approach where the participants' point of view is utilised to understand opinions and associated results (i.e., improve qualitative research results).

Based on TGD framework, the proposed system utilizes a BIM authoring tool (Autodesk Revit) as a building information provider to work with Unity3D game engine and an AMP (Apache + MySQL + PHP) database to produce an adjustable virtual reality environment throughout the building life cycle. The system allows the involvement of the end-user in refining the building emergency plan and can provide them with effective evacuation training and guidance through various commonly available mobile devices.

**Figure 3:**
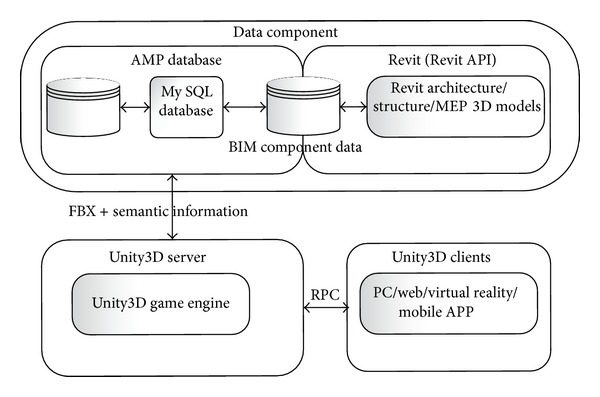
Three interconnected components of the BIM-VE.

The system architecture comprises three interconnected components (Figure 3).

- A data component contains the AMP database and Revit software. The component is controlled by an administrator to generate semantic and geometric data and store it in the database to build a two-way and dynamic information flow for real-time fire evacuation routes and training.
- A Unity server component connects Unity clients into an instance and feeds those clients the available data which is created in the data component.
- Unity clients are the interfaces that immerse the end-users into the virtual environment (as an instance) which is generated by the Unity server. Clients work in different platforms with appropriate input and output devices, that is, Windows or Mac operating systems that use high-resolution monitors with keyboard and mouse, 3D stereoscopic projector with Razer Hydra joystick, Hand Mounted Display (HMD) with Microsoft Kinect Sensor, mobile platform using iOS or Android with touch screen and built-in camera, or web based environment that allows users to connect to the server through their web-browser.

Specifically, the data component is the main part in the automation of the data transmission between the building information model (in Revit) and the serious game (in Unity3D server and clients) by means of C# based APIs connected to an AMP database. The AMP database is the central hub to collect and transfer all of the required building information, which internally develops a two-way information channel between the building information model and the serious game. It initiates the data transmission process between the building information in Revit and the required information in the AMP database. The Revit model is separated into FBX geometric model and a semantic information file. The AMP database then feeds and maps all of the building information (i.e., geometric and semantic building information) in Unity3D server based on the object IDs of the FBX model and synchronizes Unity3D server with the available clients in line with remote procedure calls (RPC). For transferring information from Unity3D to the Revit model, AMP firstly receives the altered semantic information from the virtual game environment and feeds it back to Revit. Revit then reads the changes from AMP database and compares the two semantic information sets to update the appropriate BIM components.

In addition, the Unity3D server also plays a pivotal role in the application of this two-way information flow. It bilaterally receives building information from the data component and the Unity clients, and concurrently generates the serious game environment for clients and enables updating of the building information in Revit according to the information flow in the data component. The Unity3D server consists of several component engines to create the adjustable virtual reality environment for fire emergency training and guidance. A graphic engine is critical in generating the graphical display on screen and providing the interface to load, manage, display, and animate the textured BIM components/data in the serious game. The main parts of the graphic engine are asset management, game object management, and terrain management. Asset management is responsible for the import and export of reusable game assets/packages such as shader, preferb, material, animation, and avatars. Game object management is responsible for creating new game objects with different shapes and adding particle systems, camera, GUI, light, wind, and so forth to the current scene of the serious game. Terrain management provides a handy tool to create terrain background and manage geomorphic high map effectively. The physics engine can simulate the mechanics of rigid-bodies, collisions, joints (building elements), or particle systems (smoke, fluids). Lastly, the audio engine provides the ability to generate realistic sound within the serious game.

**Figure 4:**
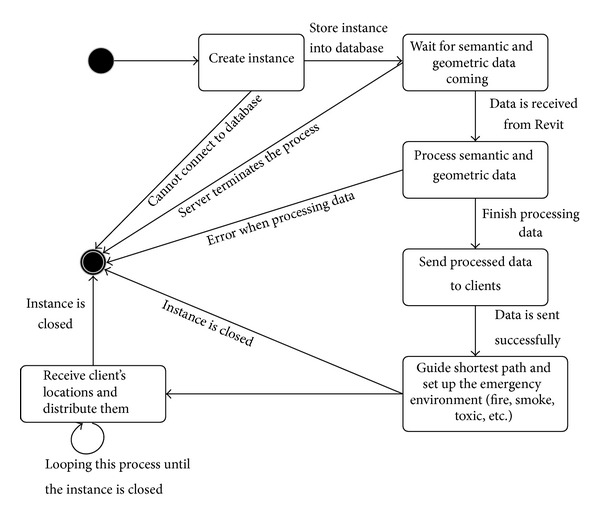
State diagram for administrator in server.

The workflow of the application on the server side is shown in Figure 4. Although there are several activities using Unity3D's built-in library for synchronization, there are some activities that are not supported or not at the layer where the Unity3D game engine can interact with them. Therefore, when the server starts up, the first procedure to implement is to create an instance in the database to store this information. Next, it waits for the semantic information to load. The loading time varies depending on the amount of building information in the BIM model. The internal process of loading data is to convert Revit building format to FBX format. Then the FBX model is loaded into the Unity server's memory and converted into our custom format, which buffers at the network layer for incoming clients to load. Finally, it is loaded and rendered on the server screen. The processed data is then sent to clients and rendered on the client side. As the process is asynchronously in the time client loading environment, an administrator on the server side can begin to calculate evacuation routes according to the updated building information in the data component. To reflect the fire circumstance, the administrator in server can also set up fire, smoke, explosion, and dangerous areas at appropriate locations.

**Figure 5:**
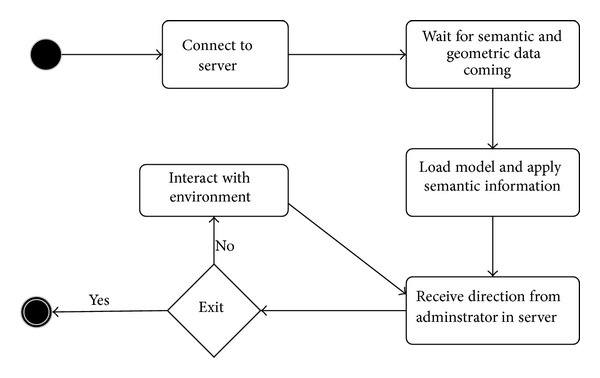
State diagram for clients.

Figure 5 shows a generic activity flow on the client side. The client first needs to connect to the server by entering the server IP address, and then it waits for the semantic and geometric data. After that, the clients automatically update the virtual environment and services based on the received building information from admin.

## 4. BIM-VE for Fire Emergency Evacuation

### 4.1. Path Finding Algorithm and Space Representation in the BIM-VE

There are some factors that humans consider often but algorithms do not fully understand such as environment based movement and time from one point to another point. Therefore, the BIM-VE integrates informative graphs in the mathematical sense—path finding algorithms to generate the shortest path on a set of vertices with edge connections based on the updated building information [27]. Space search algorithms and search space representations are two key considerations when generating real-time evacuation routes according to changing building information in the BIM-VE [28–30].

Dijkstra's algorithm, best-first search, and A∗ algorithm are three most common space search algorithms [27]. Dijkstra's algorithm works by adding all the closest not-yet-examined vertices into a representation graph from the object's starting point to the set of examined vertices. It expands outward from the starting point until reaching the goal, which is guaranteed to find a shortest path but works harder in performance and memory overhead. Conversely, the best-first search has a narrow scanning field but only considers the cost to the goal and ignores the cost of path so far, resulting in the path to goal becoming long, and tends to move forward to obstacles such as walls. The A∗ algorithm combines a heuristic approach like best-first search with formal approaches like Dijkstra's algorithm and has become the most popular choice for path finding problems and is fairly flexible in a wide range of contexts [27, 30], specifically, its knowledge-plus-heuristic cost function of notation *x* which is expressed as

(1) $$\begin{array}{c} f\left(x\right)=g\left(x\right)+h\left(x\right), \end{array}$$

where *g*(*x*) is the past path-cost function, known as the distance from the starting node to the current node *x*, and *h*(*x*) is a future path-cost function, an admissible “heuristic estimate” of the distance from *x* to the goal. Therefore, the shortest path is to keep the least-cost path from start to finish.

The success of the A∗ algorithm in shortest path generation is that it integrates space search methodology of Dijkstra's algorithm [27, 30] (i.e., *g*(*x*): higher search priority of vertices close to start point) to ensure the optimal path, with the information Best-First search explores (i.e., *h*(*x*): favouring search vertices close to the goal) to decrease the space search area. The A∗ algorithm examines the vertex *x* that has the lowest *f*(*x*) = *g*(*x*) + *h*(*x*) each time through the main loop, balancing *g*(*x*) and *h*(*x*) to move from the start point to the end point.

In the world of space search algorithms, the heuristic of the A∗ algorithm must be admissible to guarantee an optimal path, meaning that the heuristic guess of the cost from *x* to the goal must never overestimate the true cost. However, the serious game often tremendously increases the calculation speed of the A∗ algorithm at the possible expense of a slightly suboptimal path by using an overestimated heuristic guess [27, 28]. For example, a larger overestimation of the heuristic guess speeds up the calculation of the A∗ algorithm substantially at the cost of an unnoticeable suboptimal path on a large open space with random obstacles such as columns or walls. Although the suitable overestimating heuristic can enhance space search speed of the A∗, it does not mean that the underestimating heuristic is useless in the serious game. The underestimating heuristic can actually allow the A∗ algorithm to explore more nodes to get accurate and relevant search results in a complicated narrow space. Therefore, it is critical to decide what amount of overestimating or underestimating is required to balance the path optimization and calculation speed of the A∗ algorithm. The BIM-VE introduces the addition of a scale and selective *h*(*x*) in a knowledge-plus-heuristic cost function to adjust the heuristic to suit the specific problem:

(2) $$\begin{array}{c} f\left(x\right)=g\left(x\right)+\left(h\left(x\right)\times \text{scale}\right), \end{array}$$

where *h*(*x*) = |Δ*x*| + |Δ*y*| (i.e., Manhattan distance), or *h*(*x*) = max⁡⁡(|Δ*x*|, |Δ*y*|) + 0.41  min⁡⁡(|Δ*x*|, |Δ*y*|) (i.e., octile distance), or $h(x)=\sqrt{\Delta x^{2}+\Delta y^{2}}$ (i.e., Euclidean distance), in which Δ*x* is the value of distance change from current note *x* to the goal along *x*-axis and Δ*y* is the value of distance change from current note *x* to the goal along *y*-axis.

**Figure 6:**
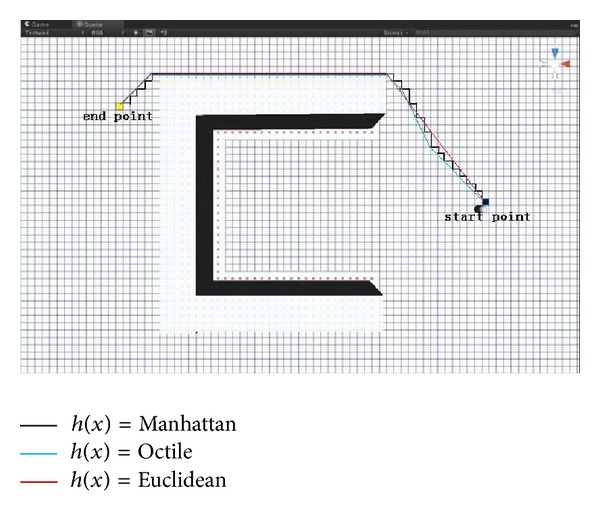
The shortest path generated by A∗ algorithm with different *h*(*x*).

If the scale is zero, the formula reduces to Dijkstra's algorithm: *f*(*x*) = *g*(*x*), which can find the optimal path at the expense of search time, because it uniformly explores outward in all directions. If the scale is larger than 1, the behaviour of A∗ algorithm is toward the behaviour of best-first search algorithm, which cannot guarantee the optimal path but finds the goal as quickly as possible. As for heuristic selection, path finding on a grid has three selective heuristics; Manhattan distance, octile distance, and Euclidean distance. Specifically, Manhattan distance does not take diagonal movement into account, which would overestimate distance. The octile heuristic (also known as Manhattan diagonal distance) assumes that only 45° and 90° are permitted for the movement, which basically corresponds to movement in the world, because it provides the most precise heuristic to use on the squared grid of a video game and therefore is the default *h*(*x*) in the BIM-VE. The Euclidean heuristic underestimates distances because it assumes the paths can take any angle but can work with a hexagonal grid to provide the most elaborate movement if necessary [27]. The BIM-VE adopting the knowledge-plus-heuristic cost function with different *h*(*x*) to calculate the shortest path from start to finish is shown in Figure 6. The black line is the shortest path generated by A∗ path finding algorithm with Manhattan distance (i.e., *h*(*x*) = Manhattan distance) and can only go following horizontal and vertical direction between neighbour grids; the blue path generated by A∗ algorithm with octile distance can move though diagonal directions between nearby grids; the red path generated by A∗ algorithm with Euclidean distance can head to any direction and connect any two grids if there is not any obstacle between them.

By altering the value of the scale and *h*(*x*), it is possible to see how A∗ behaves to suit the problem, depending on the complexity of the virtual environment. For example, A∗ can take Euclidean distance with a relatively small scale adjustment to carry out path finding in a complicated building with narrow corridors and stairs, which can guarantee that A∗ algorithm can find the shortest path using dense nodes and arbitrary directions. Since the suitable scale and *h*(*x*) have to be discovered experimentally, a user interface is provided to adjust their values in the Unity3D server to make A∗ path finding suitable to the building information in the BIM-VE.

Through the above illustrations, it is assumed that the space search algorithm is being used on a grid of some sort, where the “vertices” given to the algorithm are grid locations and the edges between the vertices are directions the shortest path could travel from a gird location. The algorithm though is only half of the picture. The space representation can make a distinct difference in the algorithm performance and shortest path quality [28, 29]. In general, the fewer vertices the space search algorithm explores, the faster it will be; the more closely the vertices match the positions that units will move to, the better the shortest path quality will be. Therefore, an appropriate space representation is required to fit into a BIM model and allow the 3D real-time path finding search in the BIM-VE [28, 29]. Generally, the three main groups of search space graphs are grid graphs, navmesh graphs, and point graphs [28, 29].

Navmesh graphs work based on triangles or polygons where each shape covers the walkable surfaces of the world. This generates very precise movement with blazing speed and low memory footprint as appropriate polygons can describe large areas with very few numbers. Similar to movement within the grid system, the navmesh graph also provides tile, edge, vertices, or a combination for path movement. However, navmesh graphs normally work on fixed search space because ever-changing space information would put a huge strain on the memory footprint leading to the possibility of a crash [28–30]. Point graphs can be built by user-placed waypoints (or “beacons”) that are linked to each other. They check the connections between waypoints to define a point of walkability. These graphs are customizable and flexible, and game designers can easily handle 3D game worlds by placing an array of points at any point in 3D space. However, they suffer from path complexity and path smooth problems because user-placed points are usually not optimized. Worse still, if the space information is changed, the user-placed points must be manually modified to avoid unrealistic path results [28–30].

**Figure 7:**
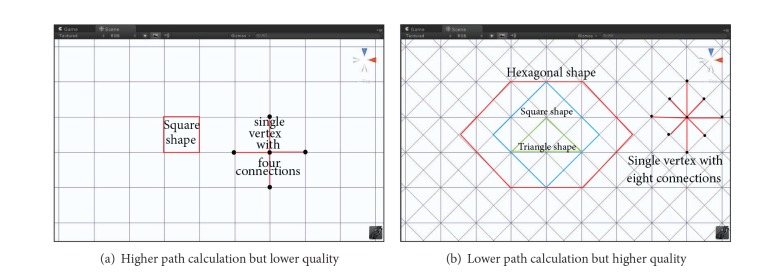
Flexible node connections to represent common shapes used by grids.

It has been shown that an A∗ algorithm working with a grid graph representation can quickly find the shortest path during runtime [31, 32]. Grid graphs represent the search space by subdividing the world into small regular shapes (i.e., tiles) and generate nodes on the shapes. Common shapes used by grids are triangular, square, and hexagonal [28–30]. The BIM-VE provides flexible connections (i.e., four or eight connections for a single vertex) to balance the shortest path calculation and quality to cover the common shapes that represent the world [32], which are shown in Figure 7. It can be seen from Figure 7(a) that a single vertex (the black dot) connects four neighbour vertices to form a square unit in the search space. It simplifies the search directions to four to get higher path calculation speed but loses path quality because the path cannot go diagonally.  Figure 7(b) shows single vertex (the black dot) connecting eight neighbour vertices forming a hexagonal, square, or triangular shape to represent the space which allows the path to go diagonally, making the path quality higher at the cost of path calculation speed. Grid graphs can quickly provide reasonable shortest paths for most spaces and can respond to runtime changes of the world graph very well because of its adaptable common shape representations. However, it cannot address the world containing overlapping areas such as a 3D building with multiple floors [32].

**Figure 8:**
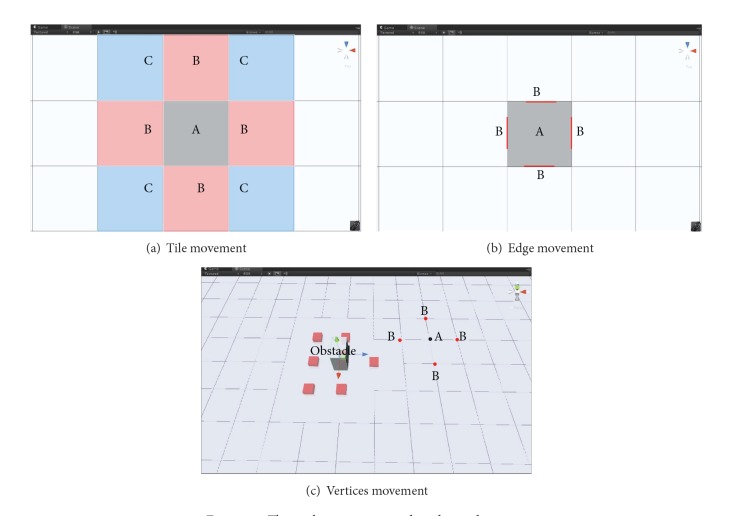
The path movement within the grid system.

Within grid graphs, there is a choice of tiles, edges, and vertices for the shortest path movement [28, 29]. Tile movement is especially useful for the virtual environment in which units only move to the centre of a tile. In Figure 8(a), the unit at A can move to any B or diagonally to C with the same or higher movement cost. If units are not constrained to grids and can move anywhere in a grid space, or if the tiles are large, edge or vertex movement would be a better choice for finding the shortest path. Compared to unit moves from centre to centre, using edge movement, the unit will move from A to B directly through one edge to the other (i.e., red line in Figure 8(b)). Obstacle corners can usually be mapped with vertices of a grid system (i.e., square red dot in Figure 8(c)). With path finding on vertices, the unit moves around an obstacle from corner to corner, producing the least wasted movement. Therefore, movement on vertices is the default for shortest path finding in the BIM-VE.

### 4.2. The Implementation of Path Finding Algorithm for Evacuation Guides

**Figure 9:**
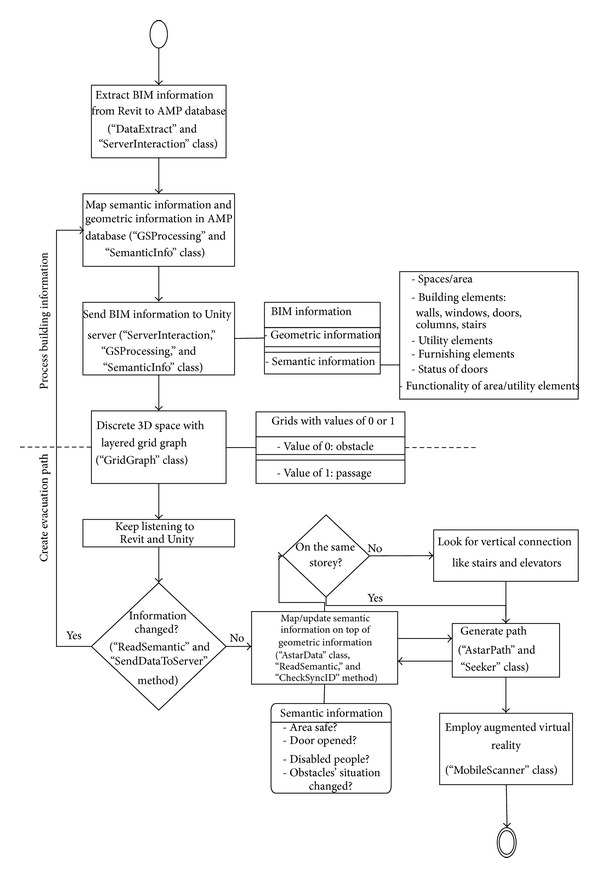
The workflow with associated classes and methods for the implementation of the path finding algorithm in the BIM-VE.

The limit of an A∗ algorithm working with the grid system for the subject of fire evacuation is that the end-users in danger may be on different floors and want to effectively find the shortest evacuation route according to ever-changing emergency information. Thus, the BIM-VE proposed utilizes an adjustable A∗ algorithm and layered grid graph to respond to a building emergency using the AMP database. The adopting workflow with main corresponding classes and methods is expressed in Figure 9.

Specifically, during the stage of processing building information, the most important two classes for automated data transferral in the data component are “DataExtract” and “ServerInteraction.” The “DataExtract” instance (objects) implements a process to read the current Revit model and write the information to FBX and semantic file. Because Revit contains all semantic information of the building design, we do not need to extract all of the information, only the useful information for fire evacuation. This useful information includes the following:

- object types: floor, wall, door, windows, fire alarm, marker, ceiling, roof, and obstacle,
- properties: unique ID, floor number of the object, height, volume, width, door status, and position marker ID number.

The “ReadSemantic” method declared in “DataExtract” class reads the property information and returns it to the “SendDataToServer” method. The “SendDataToServer” method then uses a HTTP protocol to send the results to the server and stores them in the AMP database.

The “ServerInteraction” object helps Revit interact with the Unity3D server to upload and download data. In order to interact with the server, it needs an http protocol interface that is implemented in the “HttpClient” variables of the class. The “FetchServerList” method declared in the “ServerInteraction” class downloads a list of available servers. In our current implementation, only one server can run on a machine at any one time; therefore, it always returns the single server running on the same machine. The results are stored into the “ServerIPList” and “HostList” variables (i.e., a unique ID of a server). When connecting from Revit to Unity3D server, the “SelectedServerIP” and “SelectHostID” variables are populated based on the connection, which also initialises the connection for the “HttpClient” variable.

On the other hand, “GSProcessing” and “SemanticInfo” are the most important classes which process semantic and geometric information in the AMP database. Notably, the “GSProcessing” object has several predefined variables that are critical to the BIM-VE's operation. This is because the third party library defined in the “fbxPlugin” variable to import the model is closed source. The standard size of the imported model is unknown, but the building needs to be scaled properly to allow the virtual agent to execute the path finding algorithm to pass through the open space. Therefore, the “buildingScale” variable defines the ratio size of rendering model comparing to the standard size of the imported model; the “buildingPos” variable defines the centre point of the building following the coordinates in the Unity3D Server to ensure that imported model will lay within the limits of the work area; the “HttpClient” variable defines the interaction of HTTP protocol (to download and upload data and check “SyncID” variables for synchronisation). Furthermore, the “SemanticInfo” object defines the structure of the semantic data: the “ID” variable is unique and is extracted form Revit. The “objName” variable stores the ID generated by Unity3D server for the imported object. “Type” stores the object type which is also extracted directly from Revit such as floor, wall, and door. The “pNames” and “pValues” variables store the arrays of properties extracted from Revit such as floor number, height, and volume.

In the “GSProcessing” class, the “semantics” array type is extracted at the beginning of the “ApplyingSemantics” method that is run after the “DownloadSemantic” method, which initiates a loop to check each 3D object and ID of building objects to find the appropriate “SemanticInfo” objects in the semantics array. The path finding rules will then be applied to calculate evacuation routes during the create-evacuation-path stage. For example, the A∗ path finding algorithm calculates the shortest evacuation path only based on the walkable objects (e.g., floors (without fire), (opened) doors, and (cleared) stairs) rather than unworkable objects (walls, (closed) doors, and obstacles (such as fires and dangerous areas)).

The A∗ algorithm with adjustable heuristic is distinct in this instance due to its ability to work with a layered grid graph to provide dynamic path finding within a 3D space; it checks whether the defined start and end points are on the same layer (i.e., floor) and adjusts the heuristic to ensure that the evacuation route proposed passes through walkable areas using the building semantic information. When creating shortest evacuation path, the classes that are used most in the algorithm are “AstarData,” “AstarPath,” and “Seeker.”

**Figure 10:**
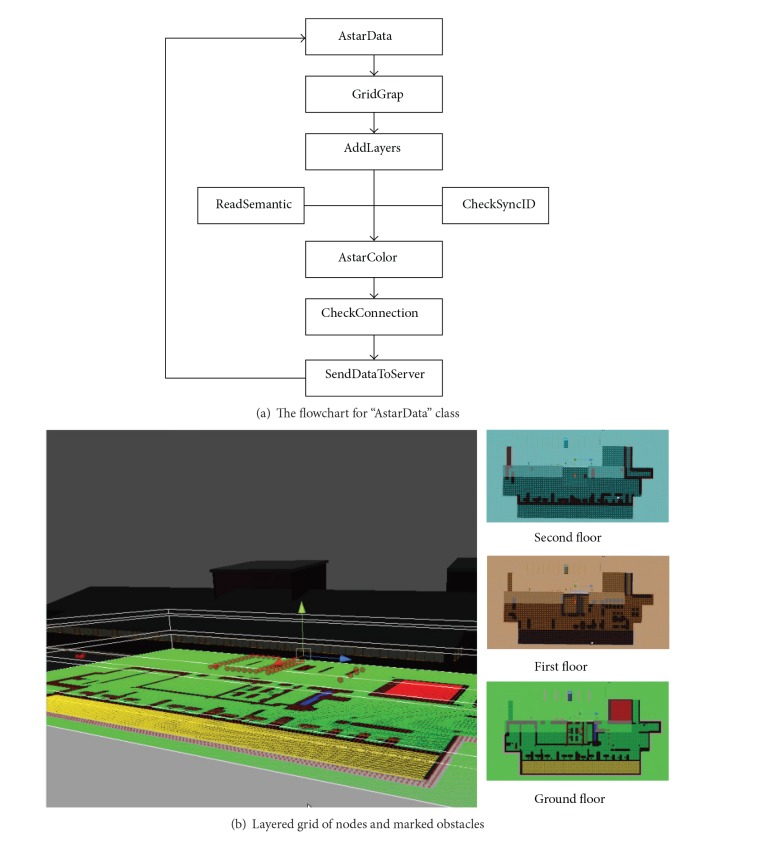
The flowchart for “AstarData” class working with its associated methods to generate and update discretized space.

During the create-evacuation-path stage (Figure 9), the “AstarData” class and associated methods work to generate and update the discretized space (workflow shown in Figure 10(a)). Specifically, the “AstarData” object stores the grid graph with the semantic information, with the instance allocated to “AstarPath.astarData” to allow access to the layered semantic grid graphs with marked obstacles. The grid generator (“GridGraph” class) in the Unity3D server generates a layered grid of nodes via the “AddLayers” method. Then, it uses “ReadSemantic” method to map the building objects and spaces with their semantic information stored in “AstarData” and marks obstacles/dangerous areas and walkable areas with different colours by “AstarColor” method. By looking for vertical connections (judged by their semantic information) such as stairs and elevators, the grid nodes that are the nearest to vertical connections in different floors are connected in *z*-axis. Then, the BIM-VE checks if layered grid nodes are connected to their neighbours and automatically links connected nodes by employing the “CheckConnection” method to create preliminary layered and coloured 3D discretized space for the shortest evacuation path.  Figure 10(b) demonstrates how grids of nodes are layered and marked for obstacles in the multifloor building. The BIM-VE marked green, brown, or blue grids of nodes for walkable area on different floors (i.e., on ground and the first and second floor separately), red nodes for obstacles, and yellow grids of nodes for closed spaces. In addition, the Unity3D server keeps checking whether the building information collected from Revit via the database and the server itself has changed, via the “ReadSemantic” and “SendDataToServer” methods, and if necessary updates the layered grid graph to create the updated discretized space at fixed time intervals. Specifically, the “SyncID” variable and “CheckSyncID” method are created to work with the “ReadSemantic” method to check if the semantic information has changed, which is critical in synchronising building information between Revit and Unity server. Herein, the semantic information flow can be used as referenced information to synchronize geometric building information between Revit and the Unity3D server. For example, doors can utilize their semantic status (i.e., open or closed) to change their geometric position to connect or block evacuation paths. Walls can be shown or disappear according to their semantic indication such as normal or broken. The checked building information in the BIM-VE includes geometric information such as dimensions of building components and semantic information such as safety of area, status of doors, materials of walls, and functionality of utilities.

The “AstarPath” class is a singleton class (i.e., only one active instance of it in the scene), which calculates evacuation routes based on information in “AstarData,” and often collaborates with the “Seeker” class that manages the path calls and path smooth for a single object in the existing A∗ shortest path library. “ShortestPath” and “Player” are the leading derived classes inherit from “AstarPath” class and “Seeker” class separately but add more attributes related to where, when, and how.

**Figure 11:**
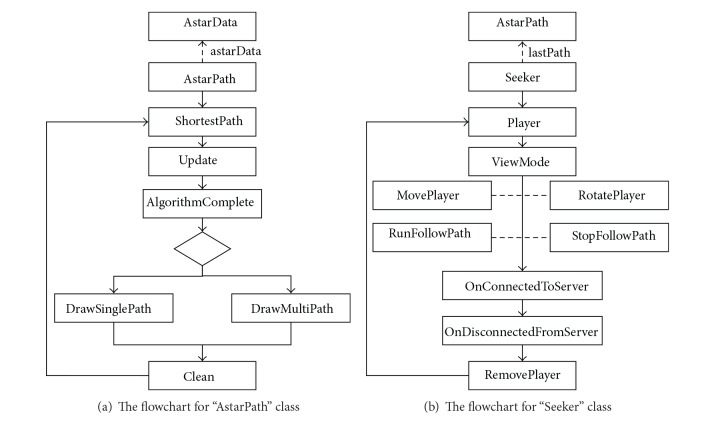
“AstarPath” class and “Seeker” class working with their associated methods to generate the shortest evacuation path and virtually follow the path.

The “ShortestPath” class is responsible for calling the existing A∗ shortest path library and putting the path finding rules on the updated layered 3D discretized space in the Unity3D server and clients through the “pathAlgorithm” interface (that is also supported by the “Seeker” object). It works with associated methods to generate real-time fire evacuation routes (Figure 11(a)). There are several important variables for “ShortestPath” to generate the evacuation path. In particular, the “lastPath” variable is a set of ordered vectors storing the latest shortest path for the server and connected clients. The “lastPoints” stores the latest points constituting the shortest path. The “pathMode” variable indicates if the current application running is single path (one start and end point) or multiple paths (one start and multiple end points). As mentioned, there are also critical methods to get and update the shortest evacuation path in the BIM-VE. The “Update” method continuously runs to construct the shortest path. When it detects that there are enough points to construct the shortest path, it will use the “pathAlgorithm” interface to run the algorithm and return the result to “AlgorithmComplete” (a callback method). The results will be stored in “lastPath” to update the current path. After synchronising the results with connected Unity3D clients, the “DrawSinglePath” or “DrawMultiPath” method (depending on “pathMode”) will draw the evacuation routes. Finally, the “Clean” method clears all the “lastPoints” and the “lastPaths” variables so that the new path can be generated.

The “Player” object is an agent to visually follow the “ShortestPath” object results, with a float value used to adjust its movement speed (Figure 11(b)). The “playerObjectID” variable stores the ID of the character being controlled by the users via Unity3D clients in the serious game. The “playerStatus” variable indicates the current movement status of the player: standing, walking, running, and so forth. The status is checked during the “Update” method and issues appropriate actions to the virtual characters. There are two “ViewMode” options available for the player to switch between; these are “freeView” and “firstPersonView.” If end-users choose the “firstPersonView,” the “MovePlayer” and “RotatePlayer” method allow end-users to use different input devices to freely control the current character and also synchronise latest movements with all other machines. Thus, the players in the Unity3D server and different clients can see the movement of each other for evacuation guidance and training. In both “ViewMode” options, “RunFollowPath” and “StopFollowPath” methods can make the character move along or stop following the shortest path if there is one, which allows them to enhance their holistic understanding of the evacuation process. The “OnConnectedToServer” method runs several initial functions to synchronise the planned shortest path between the server and clients. It also helps load the semantic models for connected clients. “OnDisconnectedFromServer” is a method that deletes the current user on all machines by using a “RemovePlayer” method if a disconnection occurs.

Lastly, the BIM-VE can further utilize augmented visualization technology to mix the virtual and real worlds when the building design is constructed. This allows general end-users to get more effective and accurate evacuation guidance and training on their mobile devices. This application currently supports Android, iOS, Blackberry, and Windows phones, meaning that nearly all smart phones and tablets with a camera can be utilised. The augmented plugin Metaio is integrated within the proposed system and is able to recognize the artificial markers to map end-users' positions between the virtual and the real. Specifically, the “MobileScanner” class implements the metaio SDK on mobile devices, which helps end-users use their mobile device camera to identify the position marker in the real world and map their locations in the virtual environment. The library from the metaio SDK conducts pattern recognition on the captured image, and, when a pattern is recognised, it converts it into an integer number called “markerID.” Metaio predefines 255 different patterns and each is linked with a unique “markerID” (from 1 to 255). A “markerID” is linked with a location that is specified by semantic data in the “GSProcessing” class. When an object is detected as a “markerID” object, the system records the coordinate and the “markerID” number.

**Figure 12:**
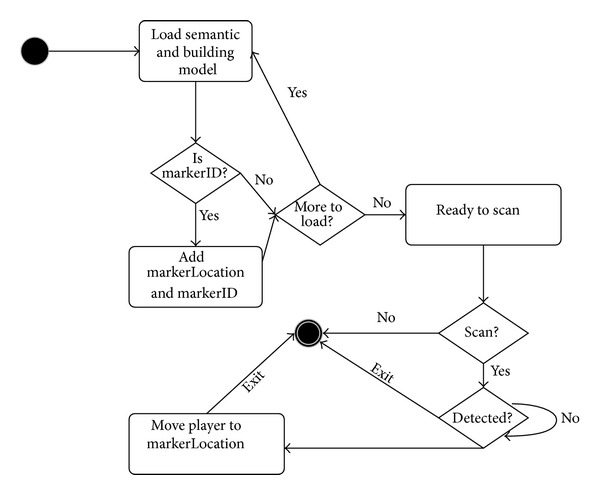
Scanning pattern on mobile devices.

Figure 12 illustrates the work flow used for locating position of the user. On application running, the “Start” method initialises the scanning process by reading a configuration file defined in metaio SDK. It then constructs empty “markerLocations” and “markerID” variables. Following this, the “Update” function continuously runs on each frame and firstly checks the camera status (i.e., turn on or turn off), and then “ScanningMarker” is called from the “Update” method to begin scanning when the camera is turned on. The “CallbackScanner” is automatically called when position markers are recognised. It works with Unity3D's built-in “getComponent” variable to get and move a player to the location of the detected “markerID” objects.

When a marker is recognized in the real world, the position of the end-users will be found in the BIM-VE. Then, the evacuation routes would use the positions of end-users as the start point to offer each end-user an intuitive evacuation guide in both a 2D minimap and a 3D model. The minimap gives an overview of the building layout and helps the user keep track of current location. In a building with multiple floors, the minimap shows the current floor map and automatically switches when the player moves to another floor. Currently, the function based on the “MiniMap” class is still limited because several important variables need manual setup, such as the textures' array to store the image of the map for each floor, and “centerPoint” to reference the 3D space centre into 2D image. The “3DToMinimap” and “minimapTo3D” are used to convert coordinates between the 3D environment and the 2D map, which keeps the positions of players in 3D space consistent with their positions on the 2D map. The “GetCurrentFloor” method is based on the player position to calculate the current floor where the player is on, and then “DrawMap” method displays the appropriate 2D map on the screen.

The BIM-VE provides end-users two modes for the evacuation service: the single-path evacuation mode and the multiple-path mode. The single-path mode helps end-users using Unity3D clients to find the recommended evacuation path from their position (recognized by position markers) to the safest point (defined by server, e.g., the nearest exit or fire rally point). In practice, evacuation paths are not unique, so the end-user using a Unity3D client can choose the multiple-path mode to find evacuation paths to all available exits. The multiple-path mode is especially useful for end-users with a disability who might be unable to follow the recommended evacuation path due to impairments such as movement limits (i.e., not easy to climb stairs or quickly pass through dangerous areas). By recognizing dangerous areas and circulation points that are not suitable for a person with a disability, these end-users can find the evacuation path that might not be the shortest but is the safest.

### 4.3. Dynamic Emergency Scenarios for Evacuation Training and Research

The application of the adjusted path finding algorithm on 3D layered discretized space can respond to the emergency environment of a building with multiple floors in real time and mix virtual and real environments to provide updated evacuation guidance on mobile devices. However, when it works with virtual reality equipment (introduced in Section 3.1) for emergency evacuation training and research, it is necessary to introduce a library approach that works with semantic building information to create dynamic fire scenarios in the Unity3D server/clients, so participants within the virtual experiment stay focused and cannot anticipate scenarios increasing reliability of experimental results.

**Figure 13:**
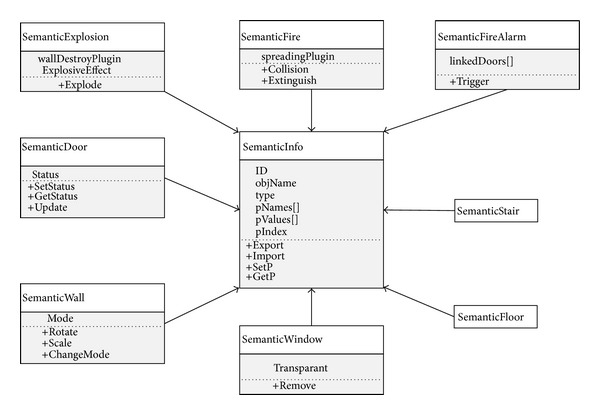
Interactive diagram between classes for dynamic emergency scenarios generation.

The library approach, where standard emergency components can be archived for reuse to create unexpected events, can not only eliminate the time wasted in repetitive data translation and optimization of rendering parts, but also add semantic information and animations to enhance the performance of the serious game. Coupled with the two-way information channel, the building information can be automatically translated into the virtual environment. However, this kind of translation does not include the definition of factors influencing fire response performance. Therefore, the library approach works on top of the bidirectional information flow to dynamically generate emergency scenarios to enhance participant's understanding of the evacuation process. Current library functions include setting up player spawn points, adding fire/toxic/smoke, display of people, running unexpected events such as explosions or wall collapses, and activating fire alarms with lights and noise. Semantic information extracted from BIM model is utilised to enhance performance of evacuation training, employing the interactive class diagram depicted in Figure 13. The “SemanticInfo” object contains the general properties of a semantic object. Working with it, specific class objects contain some special properties for handling wall collapse, automatic-shut fire doors, fire alarm activation, expanded fire, and window break events. Note that “SemanticStair” and “SemanticFloor” objects have their own class but are empty as their semantic information is currently under development for fire propagation. The “SemanticFire” object is the representation for fire, smoke, and toxic gas, which have similar properties, so it is possible to group them into one to simplify the system.

The built-in physics engine in Unity3D server is the main component to simulate the dynamic physical interaction between objects on the basis of the laws of physics such as gravity and collision. To be able to run the simulation in real time, it is inevitable that the physic performance will be simplified, because entirely correct physics calculations are not necessary and only need to “look realistic” during the evacuation training. The simulation in the evacuation training is based on the physics of rigid bodies (rigid body mechanics of objects), elastic bodies (soft body dynamics of fabrics or cloth), and object collisions (collisions between obstacles and characters). The built-in particle system simulates fire, smoke, and explosions, along with extracted material properties (semantic information) from the BIM model associated with the geometric information to simulate variable speed and dynamic fire propagation. The structural elements are split down into a finite number of smaller parts to simulate collapse events caused by explosions. Joints with up to six degrees of freedom on character bodies can be mapped with the tracking information of motion sensors to immerse participants into the 3D virtual reality environment (as described in Section 3.1).

The process to carry out fire evacuation training and research within the dynamic fire scenarios is as follows.

- The administrator within Unity3D server creates a host/session that holds building information for evacuation training and research. The two-way information channel then transfers both semantic information and geometric building information from the BIM to the serious game.
- Unity3D server then loads the appropriate emergency components with behaviour scripts through the inventory library, which works with building semantic information to create unexpected virtual fire scenarios.
- Unity3D then synchronizes fire scenarios with the Unity3D clients for evacuation training and research in the specific building.
- Before virtual experiments, participants fill in a questionnaire about their personal information and physical condition that might influence human fire response.
- The administrator introduces the building layout and how to use the BIM-VE in the virtual reality environment. The participants are told that they can quit the experiments at any time if they feel uncomfortable.
- The avatars representing participants are placed at the start point in the serious game. Based on the chosen level of participants' game experience, the suitable control version (five versions in total: desktop first person mode, desktop flight mode, 3D projector with Razer Hydra joystick, and table version) will be loaded.
- Allow participants to walk through the building in the BIM-VE, to familiarise themselves with controls and functions. Inform participants that they may communicate with other virtual characters and that environmental factors can kill their character during the evacuation.
- Restart the avatars at another spawn location. Perceived risks are gradually shown, which include people's crying, fire noise, gradual increase in fire/smoke, and other virtual characters who begin to evacuate as a group or individually.
- The administrator in Unity server then adds unexpected events such as fire and explosion to push participants to respond to the fire environment and perform the evacuation process. Additional objects like fire extinguishers are also provided to finish specific tasks for evacuation research. After finishing the evacuation process, the administrator will show specific character avatars with evacuation AI (based on the implementation of adjusted path finding algorithm on 3D layered discretized space) to simulate the efficient evacuation process. Then, the participants follow the specific avatars to carry out the evacuation, which can train their behaviour and enhance their understanding about the evacuation process.
- Lastly, a questionnaire is completed (after evacuation training) to investigate the factors that are hard to analyse through visual recording of the evacuation. The feeling and suggestion of participants are also considered.

The system keeps track of the evacuation behaviour by video recording. These recorded videos are utilized to qualitatively and quantitatively analyse the human fire response performance, in conjunction with the qualitative questionnaires at both the start and the finish.

## 5. System Testing and Evaluation

The system testing includes (1) the prototype functionality of the two-way information channel and its applications on the evacuation guidance and training, (2) the integration of virtual reality hardware and software through middleware, (3) the accuracy of evacuation simulations according to the requirements of the end-users and the ever-changing building information during a fire emergency, and (4) the effectiveness of dynamic changes of geometric and semantic building information to keep participants focused on the virtual emergency drill.

### 5.1. Scenario Based Dynamic Path Finding Test

**Figure 14:**
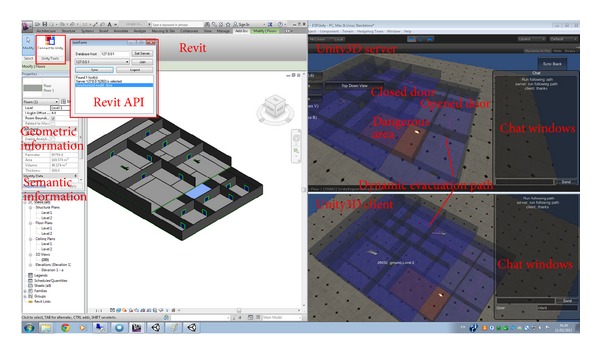
Dynamic path finding based on building information parametric in the BIM model.

The parametric nature of BIM plays a key role in rapid and accurate emergency management activities, such as real-time evacuation path guidance. However, most traditional path finding simulations for fire emergency evacuation work only in 2D space and find it difficult to respond to the ever-changing emergency circumstances due to their limitations in utilizing real-time building information. Through the two-way information flow, the adjustable path finding algorithm in conjunction with the informative layered grid graph has shown it can provide a dynamic path finding simulation in the 3D space according to the updated building information from the BIM model and the Unity3D server. The most important factors considered to influence path finding results included the building geometric layout, the door status (i.e., open or closed), the status of area (i.e., safe or dangerous), and the character status (i.e., disabled or ordinary).  Figure 14 demonstrates the dynamic path finding results based on the parametric settings of the building information in the property windows of Revit and emergency situations settings in the Unity3D server. The path finding simulation in the BIM-VE can be dynamically updated to respond to the real-time changes of the building information from both Revit and the Unity3D server and can be synchronized immediately between the Unity3D server and clients to provide accurate and timely fire evacuation guidance to the general end-users.

**Figure 15:**
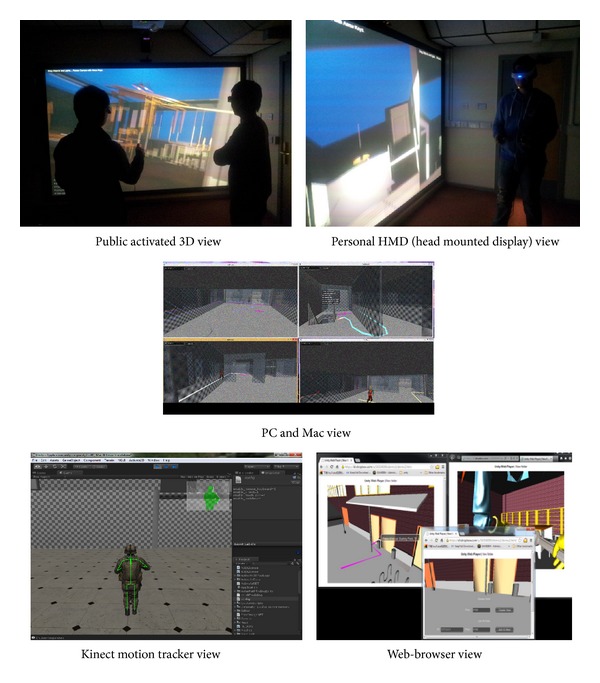
The 3D evacuation training on different platforms.

With virtual reality technology (supported by VR hardware and software), the fire evacuation path simulation can enhance the end-users' understanding of the evacuation process and make them get accustomed to the building environment and prepare them for an evacuation during a real fire disaster. Because the BIM-VE can work on multiple platforms, it is possible to engage a wide range of end-users to experience the evacuation process from an individual point of view, while walking around or through the evacuation design in different Unity3D clients (Figure 15). The virtual reality devices such as active 3D projectors, head mounted display (HMD) and Kinect for windows can immerse the local participants into the virtual fire drill to drastically improve their perception of the fire evacuation experience, than is achievable by traditional visualization methods. The local participants can further use these VR output and input devices to interact with the virtual building information model to complete specific tasks during virtual drills to deepen their understanding of the correct evacuation behaviour. In addition, network based Unity clients can support many different types of devices such as generic PCs and laptops and even web-browser based interfaces can easily connect large numbers of remote participants around the world at the same time to further enhance the service range of the fire emergency training/guidance or the research accuracy of the human fire response behaviour.

### 5.2. Evacuation Scenario Utilizing Mobile Devices Testing

The Unity3D clients of the BIM-VE support various commonly available mobile devices such as smart phones and tablets (using iOS or Android) equipped with a touch screen and built-in camera to provide the general end-user additional options for evacuation training and guidance throughout the building life cycle. It is commonly recognized that the unfamiliarity with a new or complicated building will delay the evacuation process because the evacuee is confused about how best to get out of the building during an emergency situation. The BIM-VE can transfer the building information model to the serious game for different purposes within a minute, which offers a huge potential for evacuation training during the building design stage and the evacuation guidance when the building is constructed.

**Figure 16:**
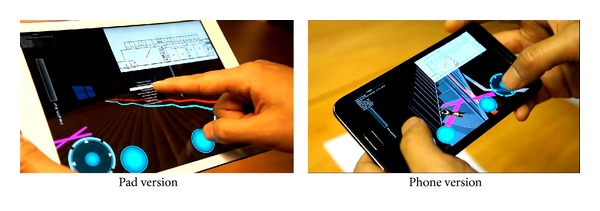
Mobile application for general end-users to carry out effective evacuation.

**Figure 17:**
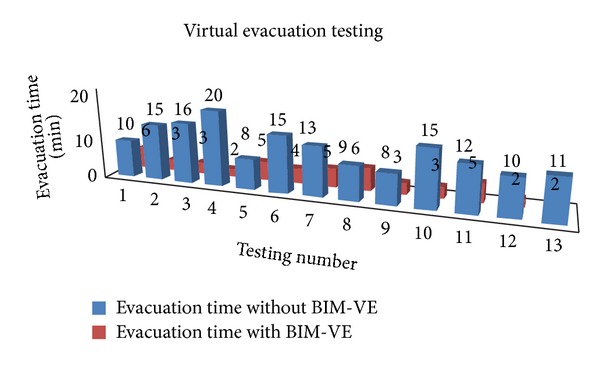
Evacuation time of virtual evacuation testing with or without mobile devices.

The west building of Cardiff University's School of Engineering was used to carry out the evacuation testing on mobile devices because the layout of the School of Engineering is complicated and often causes confusion for visitors. Several participants who are not familiar with the layout of the building were invited to attend the testing. Firstly, they were directed to walk around the engineering school to obtain the basic building information. During the wandering, they were informed of the start location (i.e., the original position to begin the testing) and the end point of the evacuation testing (i.e., an emergency exit). Then, the participants are separately guided to the evacuation start point via a route that is different from the evacuation path and required to reach the end point without assistance of mobile devices as fast as possible. It was frequently noticed that some of them became lost during the evacuation or chose the longer route to the end point. Before the second round of testing, the basic functions of the BIM-VE on mobile devices were introduced in 3D virtual space and a corresponding 2D map, to the test participants. Then, the start point and end point of evacuation test were changed (keeping the same travelling distance) in case the participants anticipated the evacuation scenario. Similar to the first round of the testing, the participants were required to evacuate from the start point to the end-point, but they moved with the support of mobile devices running the BIM-VE (Figure 16). The evacuation time with or without mobile devices is shown in Figure 17 (the results for the participants who were lost or gave up during the evacuation were not included).

**Figure 18:**
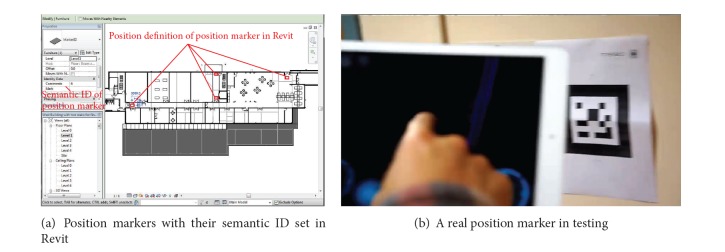
The position marker in the virtual environment and real building to help the end-users locate their position to effectively find the shortest evacuation path.

It should be noticed that the position markers that are set in Revit can help the end-users to map their positions in the real building with the 2D and 3D virtual environment during the evacuation. The BIM-VE also has other designed functions to help participants efficiently follow the recommended evacuation path generated by server such as setting current location as the begin point for the evacuation (i.e., blue evacuation route in Figure 16). The position markers with their semantic ID were mostly located on corridor junctions or places that might confuse the evacuees when trying to find the shortest evacuation routes (Figure 18(a)). In tests utilising iPhone5s, iPads, and Android phones, the marker pattern can be recognised at a distance of around 3-4 meters in normal lighting conditions and the participants position is computed in under 0.5 seconds (Figure 18(b)). The average evacuation time with assistance from mobile devices was 3.8 minutes, which provide evidence that the BIM-VE operated on mobile devices can help participants significantly decrease the time taken to evacuate the building, although its application during a real emergency situation has to be tested further.

### 5.3. Dynamic Emergency Scenario Generation Testing

**Figure 19:**
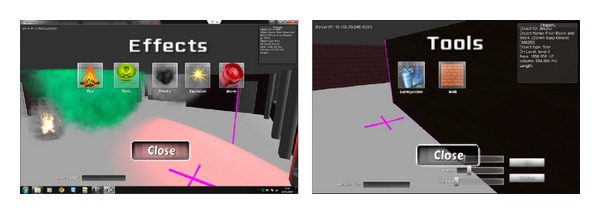
Effect and tool library to create dynamic scenarios in Unity server.

It has previously been introduced that the BIM-VE has the ability to keep participants of virtual evacuation training or human behavioral research by creating dynamic scenarios, generated by using a library based approach of building semantic information. The effect and tool library incorporated into the Unity3D server are shown in Figure 19, with the objective of increasing realism of the virtual fire disaster and eliminating any anticipation of the fire emergency scenario.

**Figure 20:**
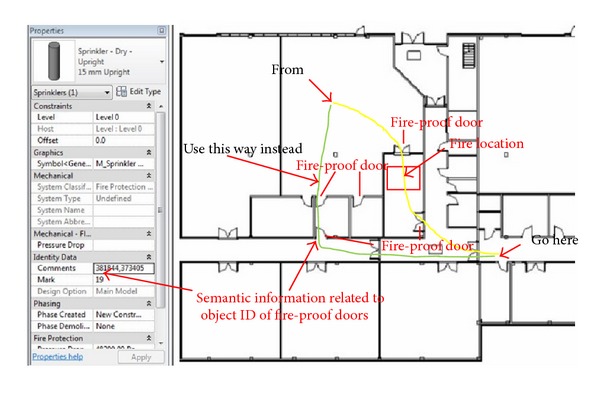
The scenario of the fire alarm works with semantic information to automatically change the fire evacuation path ((1) and (2) are fire-proof doors whose IDs are 381844 and 373405 and referenced to the fire alarm/sprinkler; yellow route is original evacuation path and the green route is the alternative path when fire alarm is activated).

**Figure 21:**
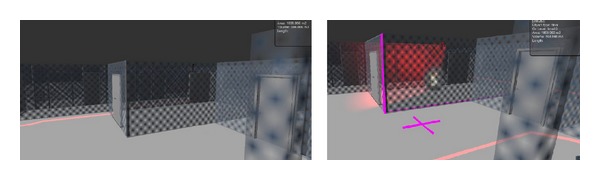
The automatic modification of evacuation path in the BIM-VE when the fire triggered the fire alarm that is referenced to fire-proof doors.

The effect library was utilized by the administrator of the Unity3D server to create unexpected events based on fire, smoke, explosion, and alarm warnings to test the participants' responses through the Unity clients during a virtual fire evacuation. These unexpected events work with building semantic information to create a vivid and dynamic building fire scenario. Take for example the fire effect on the library; it was added to the evacuation scenario in the Unity3D server with a specific time interval to create the dynamic spread of fire for the Unity3D clients. The fire spread speed is based on the building semantic information such as wall and floor's material and references to the fire engineering manual. In terms of the fire alarm, its semantic information in Revit is referenced to object IDs of fire-proof door, which control whether fire-proof doors are shut when the corresponding fire alarm is activated in fire evacuation scenario of the BIM-VE. The modification of evacuation path scenario with the semantic information of the activated fire alarm is described in Figure 20. When the spreading fire/smoke/toxic enters the detectable range of a fire alarm in the BIM-VE, the fire alarm becomes active with visual and auditory signals and shuts the referenced fire-proof door to prevent the spread of the fire while the shortest evacuation path in the Unity3D server is automatically modified and synchronized with the Unity3D clients to provide the end-users with the up-to-date evacuation training/guidance (Figure 21).

The tool library controlled by the Unity3D server aims to dynamically change the building layout and provide the participants of the Unity3D clients with specific fire-fighting equipment such as fire extinguishers to complete specific tasks. The tasks aim to explore human fire response and performance, which are mainly related to three factors: the nature of fire, the nature of the human, and the characteristics of the building. This component is still under development and will be discussed further in a following paper.

## 6. Conclusion and Future Work

This paper introduces a BIM based virtual environment (BIM-VE) aiming at improving building emergency management. The research focuses on two key factors for emergency management: (1) timely two-way information flow and its applications during the emergency and (2) convenient and simple way to increase evacuation awareness.

By utilizing the comprehensive data resources hosted in BIM, mobile devices held by building users/visitors, coupled with the help of specific tags, a real-time two-way and dynamic information flow has been successfully created and demonstrated between the virtual environment (provided by BIM and a game engine) and a real building user. The BIM-VE can create real-time evacuation routes according to the real-time location of the user.

Another research target was to provide convenient training (to building users) to increase emergency awareness. The tested scenarios have also successfully demonstrated the capability (of the developed system) to train building users, allowing them to quickly get familiar with the building and identify the right evacuation route.

The innovation lies on the seamless integration between BIM and the serious game, the semantic based smart path finding algorithm, and the leveraging of state-of-the-art technologies in practical problem solving. The system has been created and functionality has been tested; the next step is to validate its usability and actual effectiveness. A larger scale building user test is currently ongoing, and the results will be reported in further published works. In addition to this, dynamic emergency scenario generation can also be utilized to enhance reliability and validity of human fire response research; supporting virtual experiments and results are under development for further publications.

Currently, the BIM-VE works with Revit and a specific data format (e.g., FBX) to transfer building information from BIM to game, which limits its proliferation to work with other BIM software packages. As IFC (industry foundation classes) has been promoted as the de facto standard for data and process interoperability in the AEC (architecture engineering and construction) industry, one of our future works is to implement IFC interface instead of Revit API to build a universal data component to adopt different BIM packages. The robust network framework to support fast and satisfactory data transmission during emergency situations needs to be further investigated. In addition, an indoor position system (IPS) holds the potential to provide real-time indoor position services during the fire emergency, which might need to be adopted over the current marker positioning system due to the time constraints experienced by the user in a stressful emergency situation (rushing for their life). In addition, the end-users with a disability might need the safest evacuation route rather than the shortest due to their movement limits. Therefore, the specific path finding algorithm for their situation needs to be further developed.
